# Anti-proliferative effect of *Flos Albiziae* flavonoids on the human gastric cancer SGC-7901 cell line

**DOI:** 10.3892/etm.2012.771

**Published:** 2012-10-26

**Authors:** JIANMEI YUAN, WANLI LI, YUHUI TIAN, XIA WANG

**Affiliations:** 1 Departments of Chemistry; 2 Immunology; 3 Public Health and; 4 Medical Laboratory, Xinxiang Medical University, Xinxiang, Henan 453003, P.R. China

**Keywords:** *Flos Albiziae* flavonoids, proliferation, cell cycle arrest, human gastric cancer SGC-7901 cell line

## Abstract

The flavonoids found in many foods may have a protective effect against human gastric cancer. However, little information is available concerning the effects of flavonoids on the SGC-7901 cell line. Therefore, we first evaluated the effects of purified *Flos Albiziae* flavonoids (FAFs) on the proliferation of the SGC-7901 human gastric cancer cell line and investigated its possible anti-proliferative mechanisms. When SGC-7901 cells were treated with FAFs for various time periods (12–72 h) and at various doses (0–32 *μ*g/ml), cell growth decreased significantly in a time- and dose-dependent manner. Morphological observations with fluorescence microscopy and transmission electron microscopy (TEM) yielded clear evidence of cell shrinkage, formation of cytoplasmic filaments, condensation of nuclear chromatin, and cell apoptosis in the presence of FAFs. Treatment with FAFs changed the expression levels of Bcl-2, P65, Bax and caspase. The anti-apoptotic protein expression of Bcl-2 and p65 decreased gradually with the increase in FAF concentration, compared with control cells (P<0.05). FAFs contributed to the increase in Bax and caspase expression. The expression of pro-apoptotic proteins Bax and caspase were upregulated by FAFs compared with control cells (P<0.01). These results demonstrated that FAFs effectively induced apoptosis in the SGC-7901 cell line. This indicates that FAFs are likely to possess anticancer activity.

## Introduction

Gastric cancer is the most common cause of mortality and morbidity in China. A number of previous studies have evaluated the effects of various agents on gastric cancer. Mounting evidence indicates a possible role of biologically active flavonoids against many organ cancers. Human epidemiological trials have revealed an association between intake of flavonoids and reduced risk of cancer, including gastric cancer (1–7).

Flavonoids are polyphenolic compounds and display a wide range of biological activities, including anti-inflammatory and cytoprotective activities. The flavonoids have long been recognized to possess antioxidant, anti-inflammatory, antiallergic, hepatoprotective, antithrombotic and antiviral activities (1). To date, the antitumor effects of flavonoids have been commonly recognized and studied. The majority of flavonoids clearly inhibit the proliferation of cancer cells, and the effects are enhanced according to increase in concentration (2). While continuing efforts have been made to identify new molecular target-based molecules, there is an emerging interest in the chemotherapeutic application of natural substances, such as tea polyphenols and resveratrol, for cancer treatment (3). In the investigation of the cytotoxic, apoptosis-inducing, genotoxic and protective effects of the flavonoid rutin in hepatic cells, a significant association was noted between the flavonoid and NF-κB-dependent transcriptional activity (4). Flavonoids were found to influence the degree of inhibition of benzo(a)pyrene-induced DNA damage and micronuclei in HepG2 cells (5). For this reason, *Flos Albiziae* flavonoids (FAFs) have been used as an anticancer treatment. *Flos Albiziae* has also been used as a sedative in traditional East Asian medicine. The extract of *Flos Albiziae* was found to exhibit sedative activity, anticonvulsant activity and inhibitory action against the KB and Bel cell lines, and mast cell stabilizing, lipoxygenase inhibitory, hyaluronidase inhibitory, antihistaminic and antispasmodic activities (6). A highly reproducible and efficient *in vitro* shoot regeneration system was developed in a potential medicinal plant, *Albizia lebbeck*, using root explants. These results support the use of *Flos Albiziae* as a potent cytotoxic and apoptosis-inducing agent (7). However, the underlying mechanisms of anticancer activity of FAFs in SGC-7901 cells have yet to be reported. Therefore, we evaluated the effects of purified FAFs on the proliferation of the SGC-7901 human cancer cell line and further investigated the mechanisms of its antitumor ability.

## Materials and methods

### Preparation of FAFs

Dried *Flos Albiziae* was purchased from Zhangzhongjing Pharmaceutical Co. (Henan, China) and was authenticated by Professor Suping Bai at the School of the College of Pharmacy, Xinxiang Medical University, China. *Flos Albiziae* (1 kg) was gently triturated and mixed with 70% aqueous ethanol (solvent:sample = 10:1, v/w) for extraction. The mixture was refluxed in water twice for 2 h each time. The extracted solution was subsequently filtered. The collected supernatants were concentrated to 100 ml with a rotary evaporator. Next, the sample was purified by AB-8 adsorption resin and then eluted with 40% aqueous ethanol (800 ml) to elute the targets. The eluted solution was collected and concentrated to 50 ml by evaporation. This final sample was referred to as FAF. The content of flavonoids was determined with a colori-metric method described by China Pharmacopeia with minor modification. The total flavonoid content of the crude extract was determined with rutin as the reference standard. FAF was diluted with distilled water to a concentration of 50 mg/ml and stored at −70°C. The FAF solution was filtered through a 0.22-*μ*m syringe prior to use.

### Chemicals and laboratory wares

All culture media and serum were obtained from Gibco-Life Technologies. Anti-Bcl-2, anti-NF-κB P65, anti-caspase, anti-Bax, and anti-β-actin antibodies were purchased from Cell Signaling Technology Inc. All other reagents and plasticware were obtained from commercial sources. Culture dishes were purchased from Falcon Plastics. Cell lines of human cancer SGC-7901 used in our experiments were obtained from the American Tissue Culture Collection (Manassas, VA, USA).

### Cell culture and FAF treatment

All cells were cultured in Dulbecco’s modified Eagle’s medium or RMPI-1640 supplemented with 10% fetal bovine serum at 37°C in a humidified atmosphere containing 5% CO_2_. In cell proliferation experiments, cells were treated with different FAF concentrations (ranging from 0–32 *μ*g/ml) and then incubated for varying times (12, 24, 48 and 72 h). In the cell cycle analysis and apoptosis assay, cells were treated with various FAF concentrations (ranging from 0 to 32 *μ*g/ml) for 12, 24, 48, and 72 h. All experiments were performed in triplicate.

### Cell proliferation assay

The cells were harvested following trypsinization (0.025% trypsin-0.02% EDTA) and washed twice with phosphate-buffered saline (PBS). When cell density reached approximately 80% confluence, the cells were subcultured. They were then treated with FAFs. After treatment, 10 *μ*l of the labeling solution, 5 mg/ml MTT, was added to each well, and the cells were further incubated at 37°C for another 4 h. The supernatant was then discarded, and 100 *μ*l DMSO was added to each well to dissolve the resulting formazan crystals and the absorbance was recorded at 570 nm in a plate reader (Perkin Elmer). The inhibitory ratio (%) was calculated using the following equation:

$$\text{Inhibitory}\;\text{ratio}\left(\%\right)=\left[\left({\text{A}}_{\text{Control}}-{\text{A}}_{\text{Treated}}\right)/{\text{A}}_{\text{Control}}\right]\times 100\%$$

### Flow cytometric analysis of the cell cycle and apoptosis

Trypsin was used to detach the cells from the plates. Cells were then harvested in cold PBS, fixed in 70% ethanol, and stored at 4°C for subsequent cell cycle analysis. The fixed cells were incubated with propidium iodide (PI) and RNAse for 30 min at 37°C in the dark. The distributions of cells in the cell cycle were measured by flow cytometry (Becton Dickinson, FACSCalibur™). The percentages of cells in G1/G0, S and G2 phases were calculated and apoptosis analysis was performed using the CellQuest software (Becton Dickinson).

### Detection of apoptosis by fluorescence microscopy

Apoptotic cells were quantified by the fluorescence microscopy method using the Apoptotic and Necrotic and Healthy Cell Quantification kit from Biotium, Inc. (Hayward, CA, USA). SGC-7901 cells (2.5×10^5^/ml) were seeded for 48 h in 6-well plates prior to experimentation. After the cancer cells were treated with FAFs (4–32 *μ*g/ml) for 48 h, they were washed with PBS and detached from the cell culture wells with trypsin. Next, the SGC-7901 cells were centrifuged to remove the supernatant, washed with PBS and resuspended in binding buffer (100 *μ*l/sample). A combination of 5 *μ*l PI and 5 *μ*l Hoechst 33342 solutions were added to each tube. The samples were incubated at room temperature for 15 min in the dark. After staining, the cancer cells were washed with binding buffer, placed on a glass slide and covered with a glass coverslip. The stained cells were observed under a fluorescence-inverted microscope (Olympus, Tokyo, Japan). The healthy cells (stained with Hoechst 33342) emitted blue fluorescence, whereas apoptotic cells (stained with PI and Hoechst 33342) emitted red fluorescence.

### Examination by transmission electron microscopy (TEM)

The tumor cells were grown and treated with various doses of FAFs (0–32 *μ*g/ml) for 48 h in 6-well plates and were then trypsinized. A total of 5×10^6^ cells were pelleted by centrifugation at 2000 rpm for 5 min and washed three times with PBS by repeated centrifugation. The cells were then fixed in 2.5% ice-cold glutaraldehyde in 0.1 M of sodium cacodylate/1% sucrose buffer for 24 h. Next, the cells were washed in triplicate with PBS and postfixed in 1% osmium tetroxide for 60 min, encapsulated in 1% agar, and stained with uranyl acetate and phosphotungstic acid. The cells were then dehydrated in a series of graded ethanol incubated in propylene oxide and embedded in Epon 812-Araldite mixture. Ultrathin sections (50 nm) were cut using an LKL-208 ultramicrotome and placed under 200 mesh standard copper grids, followed by Hitachi H-7500 TEM analysis.

### Western blot analysis

SGC-7901 cells (2.5×10^5^/ml) were seeded for 48 h in 6-well plates prior to experimentation, and incubated with various doses (0–32 *μ*g/ml) of FAFs, as described above. After incubation, the cells were washed three times with ice-cold PBS and harvested in lysis buffer. The lysate was centrifuged at 12,000 rpm for 15 min at 4°C and the supernatant was collected. Protein concentration was determined by Bradford protein assay. Proteins (45 *μ*g/well) denatured with sample buffer were separated by electrophoresis on 12% sodium dodecyl sulfate (SDS)-polyacrylamide gels and transferred to nitrocellulose membranes (0.45 *μ*m). The membranes were blocked with 10% (v/v) dried fat-free milk in PBS containing 0.1% Tween-20 for 1 h and were incubated with anti-Bcl2, anti-NF-κB P65, anti-caspase (1:500 dilution), anti-Bax (1:500 dilution) and β-actin antibody overnight at 4°C. After washing with 1X phosphate-buffered saline Tween-20 (PBST), membranes were continuously probed with HRP-conjugated anti-mouse IgG (1:1,000 dilution) in PBS for 1 h at room temperature, then washed with PBST. The immunobinding signals were detected by a chemiluminescence method (ECL Western blotting system; Amersham Pharmacia Biotech). Relative protein expression was quantified densitometrically using the Image-Pro Plus Version 6.0 software and calculated according to the β-actin reference bands.

### Statistical analysis

All data are expressed as the means ± standard deviation. Data were analyzed by one-way analysis of variance (ANOVA) followed by Student-Newman-Keuls test for multiple comparisons. P<0.05 was considered to indicate a statistically significant difference.

## Results

### Effects of FAFs on the proliferation of the SGC-7901 human gastric cancer cell line

**Figure 1:**
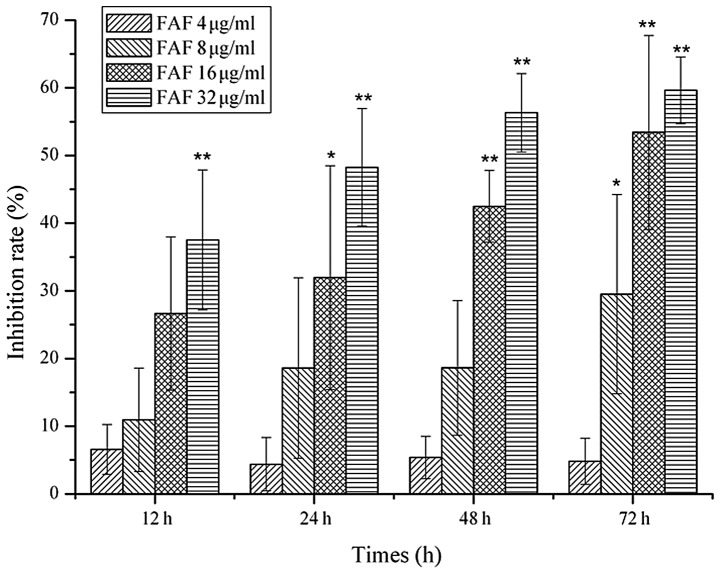
Antiproliferative activity of FAFs. SGC-7901 cells were plated in a 96-well plate and treated with various concentrations of FAFs (4, 8, 16 and 32 *μ*g/ml) for various exposure times (12, 24, 48 and 72 h). Cell proliferation was measured by MTT assay. Each group was analyzed in triplicate. ^*^p<0.05, ^**^p<0.01 compared with the lower concentration of FAFs (4 *μ*g/ml). FAFs, *Flos Albiziae* flavonoids.

MTT assay was used to investigate the antiproliferative effect of FAFs on the SGC-7901 human gastric cancer cell line at various concentrations (0–32 *μ*g/ml) and exposure times (12, 24, 48, and 72 h). As shown in Fig. 1, the cell survival percentages were reduced gradually with an increase in FAF concentration. When the SGC-7901 cells were treated with FAFs at various doses (0–32 *μ*g/ml) for various periods of time (12–72 h), cell growth decreased significantly in a time- and dose-dependent manner.

### Effects of FAFs on cell apoptosis and cell cycle progression

**Figure 2:**
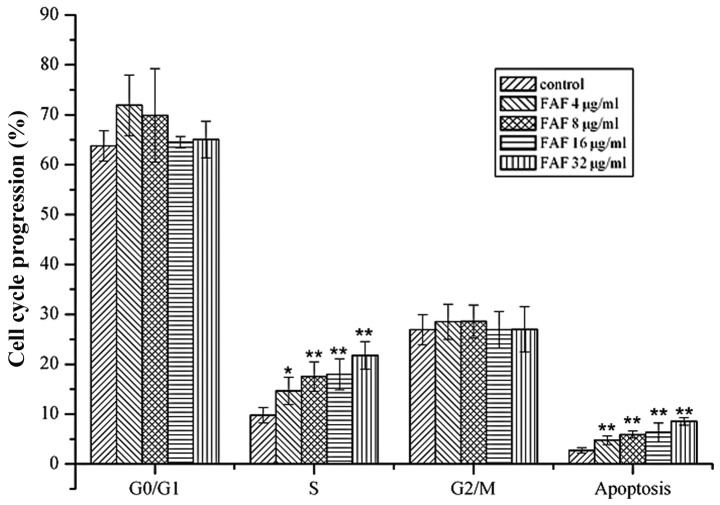
Cell apoptosis and cell cycle progression of the SGC-7901 cell line treated with FAFs. SGC-7901 cells were treated with various concentrations of FAFs (4, 8, 16 and 32 *μ*g/ml) for 72 h. Cell cycle phase distribution of the treated cells was measured by flow cytometry. Each group was analyzed in triplicate. ^*^p<0.05 and ^**^p<0.01 compared with the control group. FAFs, *Flos Albiziae* flavonoids.

The inhibition of cell growth may be a result of the induction of apoptosis that is mediated by cell cycle arrest. To determine whether the inhibitory effects of FAFs on the proliferation of the SGC-7901 cell line involved cell cycle changes, we examined the cell cycle phase distribution of the treated cells by flow cytometry. The cells were treated with various concentrations (0–32 *μ*g/ml) of FAFs for 72 h. The results showed that FAFs were capable of inducing an increase in the percentages of S-phase cells and the number of apoptotic cells (P<0.05; Fig. 2). When the concentration of FAFs was >4 *μ*g/ml, S-phase arrest in the SGC-7901 cells was significant based on the data of cell cycle distribution. In addition, the initiation of apoptosis appears to be a common mechanism of many new anticancer agents for chemotherapy.

### Apoptotic cell death was detected by fluorescence microscopy

**Figure 3:**
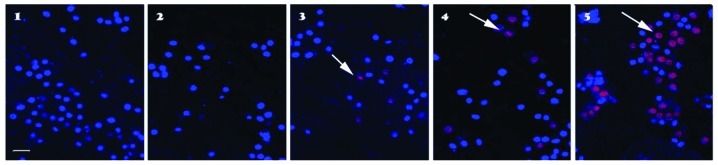
Induction of apoptosis of SGC-7901 cells following treatment with FAFs. After incubation with various concentrations of FAFs for 48 h, the cells were stained with Hoechst 33258 and PI dye and observed using fluorescent microscopy. 1, Control cells; 2, cells incubated with FAFs (4 *μ*g/ml); 3, FAFs (8 *μ*g/ml); 4, FAFs (16 *μ*g/ml) and 5, FAFs (32 *μ*g/ml). Apoptotic cells were detected and visualized by fluorescence microscopy using Hoechst 33342 and PI staining. Healthy cells (stained with Hoechst 33342) emitted blue fluorescence, and apoptotic cells (stained with Hoechst 33342 and PI) emitted red fluorescence (arrows). Scale bar, 100 *μ*m. FAFs, *Flos Albiziae* flavonoids; PI, propidium iodide..

In order to further evaluate whether the cell growth inhibition was caused by apoptosis, SGC-7901 cells were stained with Hoechst 33258 and PI dye following treatment with various concentrations of FAFs for 48 h. As presented in Fig. 3, control cells showed homogeneous blue fluorescence and an intact nuclear structure. Conversely, following treatment with FAFs, the cells exhibited typical characteristics of apoptosis, such as red fluorescence emission and nuclear chromatin condensation. Overall, such apoptotic features became intensified as the FAF concentrations increased. The cell morphology results suggested that FAF-reduced cell viability was related to apoptosis.

### Ultrastructure of tumor cells under TEM

**Figure 4:**
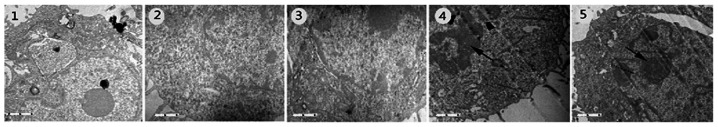
Induction of apoptosis of SGC-7901 cells following the treatment with FAFs. After incubation with various concentrations of FAFs for 48 h, the cells were examined using TEM. 1, Control cells; 2, cells incubated with FAFs (4 *μ*g/ml); 3, FAFs (8 *μ*g/ml); 4, FAFs (16 *μ*g/ml) and 5, FAFs (32 *μ*g/ml). The cells treated with FAFs (16–32 *μ*g/ml) showed typical apoptotic phenomena and apoptotic bodies has been found (arrows). Scale bar,1 *μ*m. FAFs, *Flos Albiziae* flavonoids; TEM, transmission electron microscopy.

Cell morphological changes were examined to observe the effect of FAFs on SGC-7901 cells by TEM. The control and FAF (4–8 *μ*g/ml) cell groups presented a marked viability; the cells showed nearly no change, as revealed by Hitachi H-7500 TEM. As shown in Fig. 4, when the FAF concentration was raised to 8 *μ*g/ml, there was a slight reduction in cell numbers. However, following incubation with a dose of 16 *μ*g/ml FAFs for 48 h, the characteristic features of the SGC-7901 cells were notably changed; shrinkage, cytoplasm condensation and formation of cytoplasmic filaments were noted. At the higher doses of FAFs, these characteristics were more pronounced and the cells displayed partial detachment. Here, some substances in the cytosol appeared to be released into the media and aggregation of rounding dead cells became evident. In the FAF (16 and 32 *μ*g/ml) groups, there were more apoptotic cells and apoptotic bodies than were noted in the FAF (4–8 *μ*g/ml) groups.

### Changes in Bcl-2, p65, Bax and caspase protein expression in the SGC-7901 cell line

**Figure 5:**
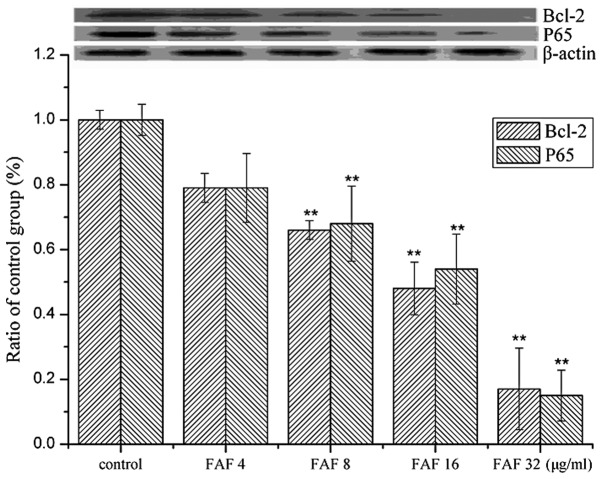
Western blot analysis for the expression of Bcl-2 and P65. Cells were treated with various concentrations of FAFs (4, 8, 16 and 32 *μ*g/ml) for 48 h. Relative protein expression was quantified densitometrically using the Image-Pro Plus Version 6.0 software and calculated according to the β-actin reference bands. Reported values are the means ± SD (n=3). ^**^p<0.01 compared with the control group. FAFs, *Flos Albiziae* flavonoids.

**Figure 6:**
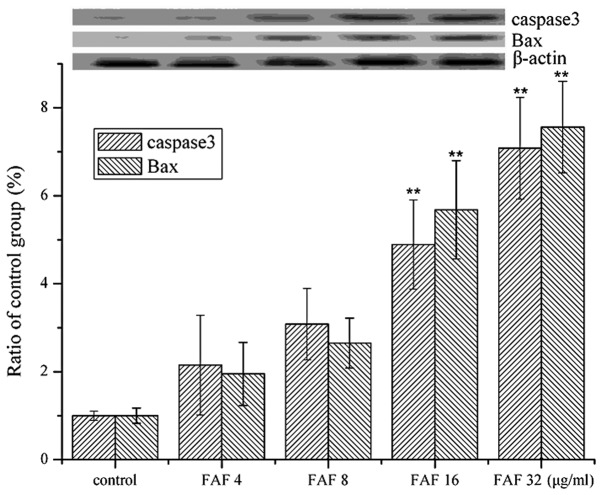
Western blot analysis for the expression of caspase 3 and Bax. Cells were treated with various concentrations of FAFs (4–32 *μ*g/ml) for 48 h. Relative protein expression was quantified densitometrically using the Image-Pro Plus Version 6.0 software and calculated according to the β-actin reference bands. Reported values are the means ± SD (n=3). ^**^p<0.01 compared with the control group. FAFs, *Flos Albiziae* flavonoids.

To clarify the mechanisms of apoptosis caused by FAFs in the SGC-7901 cell line treated with various concentrations of FAFs (4, 8, 16 and 32 *μ*g/ml) for 48 h, we assayed the protein expression of an apoptosis mediator. To ensure equal loading of proteins in all the samples, β-actin control was used. After the SGC-7901 cells were treated with FAFs, western immunoblotting was used to analyze the expression of Bcl-2, P65, Bax and caspase. Bcl-2 and P65 are proteins considered as inhibitors of apoptosis. Bax and caspase are pro-apoptotic proteins. Fig. 5 shows the effect of FAFs on the protein expression of Bcl-2 and p65 in SGC-7901 cells. The anti-apoptotic protein expression of Bcl-2 and p65 decreased gradually with the increase in FAF concentration compared with the control cells (P<0.05). FAFs contributed to the increase in Bax and caspase expression. Fig. 6 shows the effect of FAFs on the protein expression of Bax and caspase in SGC-7901 cells. The expression of the pro-apoptotic proteins Bax and caspase was upregulated by FAFs compared with the control cells (P<0.01). These results demonstrate that FAFs effectively induced apoptosis in the SGC-7901 cell line.

## Discussion

In recent years, phytochemicals or their analogs have been recognized as a new prevention and therapeutic approach for human cancers. A large number of chemopreventive agents existing in natural products have been evaluated using various experimental models. Flavonoids, a major class of non-nutritive dietary compounds, are present in most plants, and possess various bioactive properties (8,9). The pharmacological properties of flavonoids are diverse: antioxidant, vasodilatory, anticarcinogenic, anti-inflammatory, antibacterial, immune-stimulating, antiallergic and antiviral activities have all been reliably reported (10–12). Therefore, flavonoids are thought to be beneficial compounds for cancer chemoprevention. A number of them have progressed to early clinical trials. More recently, the focus has been directed towards molecular targeting of chemopreventive agents to identify the mechanism of action of these newly discovered bioactive compounds, and drug combinations with resveratrol have been revealed to impart greater suppressive activity than either agent alone. This supports the hypothesis that development of novel combination therapies/chemoprevention using dietary agents will be more beneficial against cancer (13,14). Some of the most promising and well-documented are turmeric (curcumin), resveratrol, silymarin, EGCG and genistein. These dietary agents have been found to modulate multiple signaling and apoptotic pathways in tumor cells, elucidating their role in prevention and treatment of cancer (15,16).

FAFs have a variety of biological functions, but to date there has been little information to further verify them. When SGC-7901 cells were treated with FAFs at various doses (0–32 *μ*g/ml) and for various periods of time (12–48 h), cell growth decreased significantly in a time- and dose-dependent manner. We first examined the concentration- and time-response effects of FAFs on the SGC-7901 human cancer cell line. The results showed that FAFs inhibited the growth of those cancer cells in a concentration- and time-dependent manner. This was consistent with previous reports stating that flavonoids result in the inhibition of proliferation of human hepatocellular carcinoma cells in a dose- and time-dependent manner (17,18).

Apoptosis plays an important role in controlling cell numbers in a number of physiological and growth processes, in tissue homeostasis, and in the regulation of the immune system, whereas insufficient apoptosis is an integral part of cancer development (19). Recently, inducers of apoptosis have been used in cancer therapy, and activation of apoptotic pathways is a key mechanism by which cytotoxic drugs kill tumor cells, which is now considered as an important method of assessment for the clinical effectiveness of many antitumor drugs (20). In the present study, we showed that FAFs induce apoptosis in the SGC-7901 cell line. To confirm apoptosis in the SGC-7901 cells, the morphological changes in the cells were examined by TEM and fluorescence microscopy. The results showed that the cells treated with FAFs exhibited apoptotic features. The data strongly indicate that the pharmacological effects of FAFs *in vivo* occurred through the induction of apoptosis in the cells examined.

To clarify the mechanisms of apoptosis caused by FAFs in the SGC-7901 cell line, western blot analysis was used to analyze the expression of Bcl-2, P65, Bax and caspase. Bcl-2 has previously been shown not only to inhibit apoptosis, but also to exert growth inhibitory activity on tumor cells, and to prevent their re-entry into the cell cycle from a G0-like state by distinct mechanisms (21). Bcl-2 protein also induces erythroid differentiation, which partially sensitizes the cells to drug-induced differentiation and apoptosis by expressing Bcl-xS. In addition, the ratio between death repressors (Bcl-2, Bcl-xL) and death promoters (Bcl-xS) in the Bcl-2 family will determine the cell’s fate upon diverse stimuli (22). The current study has demonstrated for the first time that the inhibitory effect of FAFs on cancer cells occurs due to the modulation of cell cycle progression. Higher concentrations of FAFs markedly inhibited the growth of cells by S-phase arrest and promoted increased tumor cell apoptosis. As has been established, cell cycle progression is regulated by several different cyclin-dependent kinases, which are activated through binding with various types of cyclins. The antiapoptotic proteins Bcl-2 and P65 were downregulated by FAFs, whereas the proapoptotic proteins Bax and caspase were upregulated, followed by induction of apoptosis. These results demonstrated that FAFs directly inhibited the growth of cells via the S-phase signaling pathway, indicating that the antitumor activity of FAF compounds was at least partially due to the inhibition of this pathway. The induction of apoptosis by FAFs may act as a possible strategy for cancer chemotherapy.
